# Geriatric rehabilitation of stroke patients in nursing homes: a study protocol

**DOI:** 10.1186/1471-2318-10-15

**Published:** 2010-03-27

**Authors:** Monica Spruit-van Eijk, Bianca I Buijck, Sytse U Zuidema, Frans LM Voncken, Alexander CH Geurts, Raymond TCM Koopmans

**Affiliations:** 1 Department of Primary and Community Care, Centre for Family Medicine, Geriatric Care and Public Health, Radboud University Nijmegen- Medical Centre, Geert Grooteplein 21 Nijmegen 6525 EZ, the Netherlands; 2 SVRZ, Koudekerkseweg 143, Middelburg 4335 SM, the Netherlands; 3 De Zorgboog, Roessel 3, Bakel 5761 RP, the Netherlands; 4 Department of Rehabilitation, Nijmegen Centre for Evidence Based Practice, Radboud University Nijmegen- Medical Centre, Geert Grooteplein 21, Nijmegen 6525 EZ, the Netherlands

## Abstract

**Background:**

Geriatric patients are typically underrepresented in studies on the functional outcome of rehabilitation after stroke. Moreover, most geriatric stroke patients do probably not participate in intensive rehabilitation programs as offered by rehabilitation centers. As a result, very few studies have described the successfulness of geriatric stroke rehabilitation in nursing home patients, although it appears that the majority of these patients are being discharged back to the community, rather than being transferred to residential care. Nevertheless, factors associated with the successfulness of stroke rehabilitation in nursing homes or skilled nursing facilities are largely unknown. The primary goal of this study is, therefore, to assess the factors that uniquely contribute to the successfulness of rehabilitation in geriatric stroke patients that undergo rehabilitation in nursing homes. A secondary goal is to investigate whether these factors are similar to those associated with the outcome of stroke rehabilitation in the literature.

**Methods/Design:**

This study is part of the Geriatric Rehabilitation in AMPutation and Stroke (GRAMPS) study in the Netherlands. It is a longitudinal, observational, multicenter study in 15 nursing homes in the Southern part of the Netherlands that aims to include at least 200 patients. All participating nursing homes are selected based on the existence of a specialized rehabilitation unit and the provision of dedicated multidisciplinary care. Patient characteristics, disease characteristics, functional status, cognition, behavior, and caregiver information, are collected within two weeks after admission to the nursing home. The first follow-up is at discharge from the nursing home or one year after inclusion, and focuses on functional status and behavior. Successful rehabilitation is defined as discharge from the nursing home to an independent living situation within one year after admission. The second follow-up is three months after discharge in patients who rehabilitated successfully, and assesses functional status, behavior, and quality of life. All instruments used in this study have shown to be valid and reliable in rehabilitation research or are recommended by the Netherlands Heart Foundation guidelines for stroke rehabilitation.

Data will be analyzed using SPSS 16.0. Besides descriptive analyses, both univariate and multivariate analyses will be performed with the purpose of identifying associated factors as well as their unique contribution to determining successful rehabilitation.

**Discussion:**

This study will provide more information about geriatric stroke rehabilitation in Dutch nursing homes. To our knowledge, this is the first large study that focuses on the determinants of success of geriatric stroke rehabilitation in nursing home patients.

## Background

According to the World Health Organization, 15 million people worldwide suffered a stroke in 2004 [1]. It has been reported that the mean stroke incidence rate in Western countries is 94 per 100.000 person years [2]. Although men are more often affected than women due to a younger age of onset, this gender difference becomes smaller with increasing age [3]. Stroke incidence typically increases with age and, due to the ageing of the population, stroke incidence rates are expected to rise. High age and low level of physical endurance, due to significant comorbidity, are characteristic of the geriatric stroke population. Although rehabilitation after stroke is an important activity in many rehabilitation centers worldwide, most geriatric stroke patients are probably not admitted to these centers and, thus, do not participate in intensive rehabilitation programs [4]. These patients may be referred to nursing homes or skilled nursing facilities (SNF) that provide adapted rehabilitation programs combined with residential care, whereas others may not receive any formal type of multidisciplinary rehabilitation at all. As a result, geriatric stroke patients are greatly underrepresented in outcome studies and factors associated with the successfulness of their rehabilitation are largely unknown.

Few studies have dealt with the influence of comorbidity and age on the outcome of stroke rehabilitation. Atalay and Turhan [5] found that elderly stroke patients (older than 65 years of age) were less likely to be successfully rehabilitated despite similar Functional Independence Measure (FIM) scores on admission, compared to patients younger than 65 years. Yet, comorbidity and age were not associated with prolonged length of stay in the rehabilitation center. In the same vein, Fischer et al. [6] found that comorbidity and age did not uniquely contribute to predicting length of hospital stay. On the other hand, there is evidence that comorbidity and age are important factors in determining functional outcome after stroke [7]. Several additional studies have emphasized the importance of age for functional outcome after stroke, but estimates of the true impact of age seem to vary greatly. Whereas some studies reported a relatively small influence of age [8,9], other studies found that very old age, defined as 85 years and older, was a consistently strong predictor of poor outcome [10].

Interestingly, Teasell et al. [4] have reported that rehabilitation in 'lower band' patients recovering from severe stroke, who were considered inappropriate for conventional inpatient rehabilitation programs, may still be quite successful in terms of gain in independency of self-care and ambulation. However, although the patients were on average 72 years of age, this study did not specifically focus on geriatric rehabilitation and did not examine the influence of comorbidity or age on rehabilitation outcome. Several other studies have shown that a substantial number of stroke patients that receive rehabilitation in SNFs or nursing homes can be successfully discharged to the community [11-13]. The probability of discharge greatly depends on individual rehabilitation potential, which is related to stroke severity and physical capacities. Besides, it appears that admission to SNFs increases the likelihood of successful rehabilitation in terms of discharge to the community [11,12].

In general, many studies have investigated the clinical, biological and demographic factors associated with the outcome after stroke [4-10,14-25]. A large number of such factors has been associated with the outcome after stroke rehabilitation (table 1), but probably many of these factors are interrelated. This implicates that the unique contribution of these factors to stroke outcome, corrected for association with other factors, still has to be determined in order to be of value for clinical prediction in daily practice. In short, initial disability and age seem to be the most promising predictors of long-term activities of daily living (ADL) and discharge destination after rehabilitation.

**Table 1:** Factors associated with stroke outcome disability and discharge destination in the literature

Outcome	Factors associated with outcome
**ADL scores**	
**FIM**	- Initial FIM, age [8,9]
**BI**	- Initial BI [14]
	- Initial NIHSS, age, premorbid disability, DM, infarct volume [15]
	- Trunk Impairment Scale, static sitting balance [16]
	
**Discharge destination**	
	- Age, incontinence [18]
	- initial FIM, age [17]
	- premorbid social support, FIM bowel, age, CMSA leg, type of premorbid accommodation [19]
	- initial MMSE, premorbid living with relatives [8]
	- discharge BI, LOS, age [20]
	- Initial FIM, age, male gender [4]
	- swallowing disorder [21]

FIM functional independence measure, BI barthel index, NIHSS

national institute of health stroke scale, DM diabetes mellitus,

CMSA Chedoke-McMaster stroke assessment, LOS length of stay

Against this background, the primary goal of this study is to assess the factors that uniquely contribute to the successfulness of rehabilitation in geriatric stroke patients that undergo rehabilitation in nursing homes. Functional outcome is primarily assessed by discharge to an independent living situation and, secondarily, by various functional scales. A secondary goal is to investigate whether the factors that are uniquely associated with successfulness of rehabilitation in this geriatric population are similar to those associated with the outcome of stroke rehabilitation in the literature. To this end, we have set up a multicenter study in 15 nursing homes in the Southern part of the Netherlands. All participating nursing homes are selected based on the existence of a specialized stroke rehabilitation unit and the provision of dedicated multidisciplinary care. To our knowledge, this is the first study that focuses on the determinants of success of geriatric rehabilitation in nursing home patients.

## Methods/Design

### Study design

This prospective study is part of the Nijmegen Geriatric Rehabilitation in AMPutation and Stroke (GRAMPS) study and comprises three measurements. Baseline data (T0) are collected within two weeks after admission to the nursing home. Patients and disease characteristics, functional status, cognition, behavior and caregiver information are registered (table 2). The first follow-up (T1) is at discharge from the nursing home, and focuses on functional status and behavior. Successful rehabilitation is defined as discharge from the nursing home to an independent living situation within one year after admission. The second follow-up (T2) is at three months after discharge in patients who rehabilitated successfully and focuses on functional status, behavior and quality of life.

**Table 2:** Research instruments

	Instrument	T0	T1	T2
**Patient**	Patient characteristics	X		
	Co-morbidity: Charlson Index	X		
	Medication list	X	X	
				
**Functional status**	Motricity index Arm and Leg*	X		
	Trunk control test*	X		
	Trunk impairment scale	X		
	Barthel index*	X	X	X
	Social activity: Frenchay activities index*	X		X
	One leg standing balance	X	X	X
	Frenchay arm test*	X	X	X
	Berg Balance scale*	X	X	X
	Functional Ambulation Categories*	X	X	X
	10 m walking speed*	X	X	X
	Water swallowing test*	X		
				
**Cognition**	Mini Mental State Examination	X		
	Star cancellation test	X		
	Hetero anamnestic cognition test	X		
	Apraxia test	X		
	Communication: SAN score*	X		
				
**Behavior**	Neuropsychiatric inventory questionnaire	X	X	X
	Neuropsychiatric inventory Nursing Home	X	X	
**Quality of life**	Global depression scale 8	X	X	X
				
**Caregivers**	RAND 36 version 2			X
				
	Social situation	X	X	X
	COOP WONCA	X		
	Caregiver strain index*			X

*: test recommended by the Netherlands Heart Foundation

SAN stichting afasie Nederland (Dutch Aphasia Foundation), COOP WONCA The

Dartmouth COOP Functional Health Assessment Charts/WONCA

Data collection has started in January 2008, and will end in July 2010.

### Patients

All patients who are consecutively admitted to one of the specialized rehabilitation wards of the 15 participating nursing homes are eligible to participate in this study. No other inclusion criteria were applied. Inability to give informed consent is an exclusion criterion. All participating nursing homes collaborate in the Nijmegen University Nursing Home Network of the Radboud University Nijmegen Medical Center. After admission patients are provided with oral information from the treating physician or nurse. In addition, all patients and their caregivers receive written information about the study. The patients indicate themselves whether they are interested to participate. The attending physician judges the legal capacity of his/her patients. In the case of doubts he/she consults the caregivers. In addition, the GRAMPS website http://www.gramps.nl provides extra information for interested patients and their caregivers.

### Ethical approval

This research protocol was presented to the medical ethics committee of the district Nijmegen- Arnhem, the Netherlands. Ethics approval was not deemed necessary, because the design is observational and because legally incapable patients are excluded.

### Assessment instruments

Data are collected by the multidisciplinary teams working in the participating nursing homes. Each discipline has the obligation to perform specific assessments. The selected outcome measures have been selected based on previously established reliability and validity or based on recommendations by the Netherlands Heart Foundation guidelines for stroke rehabilitation (table 2)[26].

#### • Patient characteristics

General patient characteristics as well as disease characteristics, medication lists, and information about comorbidity, using the Charlson Index (CI), are registered. The CI comprises 19 categories of diagnoses from the International Classification of Diseases, (9th revision Clinical Modification ICD-9CM) and is based on a set of risk factors for one-year mortality risk [27]. The CI contains a weighted index for each disease at which the score is a significant predictor of one-year survival. One-year mortality rate for the different scores are: "0" 12%, "1-2" 26%, "3-4" 52% and ">5" 85%.

#### • Functional status

The Barthel Index (BI), modified by Collin et al. in 1988 [28], measures dependency in activities of daily living (ADL). The BI is a valid and reliable instrument in stroke research [28-31]. The total score ranges from 0-20, with 20 representing complete functional independence. The Frenchay activities index (FAI) is used for assessment of extended ADL. The FAI [32] scores the actual activities undertaken by patients and can be divided in three domains: domestic housework, indoor activities and outdoor activities. The 15-item questionnaire is a reliable and valid instrument for measuring functional outcome in stroke patients [33,34]. Even proxies give reliable information about FAI items [35,36].

The Frenchay Arm Test (FAT) is used to evaluate arm function after stroke. The patient is asked to perform five activities with his affected arm, for which he receives one point if successfully complete. The FAT is a valid and reliable instrument for use in stroke research [37].

The Motricity Index [38] is used to evaluate motor impairment of the limbs. Six movements, divided in arm and leg movements, are observed. Three scores can be measured: arm score, leg score and side score. Both arm and leg scores have good criterion validity and are reliable if used by different observers [39-41].

Item three of the Trunk Control Test (TCT) is used to assess static sitting balance: sitting in a balanced position on the edge of the bed for at least 30 seconds, with the feet above the ground. The Trunk Impairment Scale (TIS), developed by Verheyden and colleagues [42], evaluates motor impairment of the trunk after stroke. TIS takes movement and coordination as well as static sitting balance into account. The TCT and TIS both show good validity and reliability [40,42].

The Berg Balance Scale (BBS)is an ordinal 14 item scale (0-56 points) developed by Berg et al. [43] to measure balance in stroke patients. Validity and reliability of the BBS is good [44-47], however the scale is not suitable for patients with very severe impairments, who cannot maintain a balanced sitting position [44]. Ceiling effects have also been described by Mao [44] at 90-180 days post stroke. The one- leg- standing balance test, first used by Schoppen et al. [48], is used to assess standing balance on the unaffected leg.

The Functional Ambulation Categories (FAC) [49] is a measure of the (in)dependency of gait. The FAC is an ordinal six-point scale with 0 indicating total dependency for walking and 5 indicating independent walking. The use of a walking device is allowed. Berg et al. [43] found high correlations between the BBS and FAC scores.

The Ten-Meter-Walking-Speed test (TMWS-test) times the walking speed along a distance of ten meters and can be performed at a comfortable or maximum walking speed [50]. Because the comfortable walking speed seems to be more responsive to functional recovery after stroke [51] and because the maximum walking speed can be estimated by multiplying comfortable walking speed by 1.32 [52], the TMWS- test is performed at comfortable walking speed, only by patients with a FAC score of 3 or higher.

The water swallowing test [26] is a simple bed-side test and resembles the water swallowing test proposed by Smithard and coworkers [21]. After drinking three spoons of water safely, half a glass of water is given to the patient. The patient fails in case of signs of choking. The speech therapist assesses food consistency after the patient safely drinks the water.

#### • Cognition

The Mini- Mental- State- Examination (MMSE), developed by Folstein and McHugh [53], is a screening instrument for cognitive impairment, and has a fair reliability and construct validity, with a high sensitivity for moderately-severe cognitive impairment and a lower sensitivity for mild cognitive impairment [54]. It comprises items testing orientation, attention, memory, language and constructive abilities. Bottom and ceiling effects have been described [55]. An important bias in using the MMSE in stroke research is the extensive use of language, which leads to unreliable results in aphasic patients. For this reason, we will not use the MMSE in patients with severe aphasia. The Hetero-Anamnestic- Cognition list (HAC list), derived from the MMSE by Meijer in his AMDAS study [56], is used to explore the presence of premorbid cognitive disabilities. The proxy, preferably a partner if present, is asked a few simple 'yes' or 'no' questions concerning orientation, attention and calculation, language, memory, and executive skills. Severity is judged on the basis of need of assistance or professional therapy required.

The Star Cancellation Test (SCT), an item of the Behavioral Inattention Test (BIT) [57], is a screening instrument for detecting unilateral visuospatial neglect. The SCT consists of 52 large stars, 13 characters, 10 words, and 56 small stars. All small stars are to be eliminated. The researcher gives a demonstration by crossing out the two small stars in the middle. The cut-off point is 52 [57]. Rough scores can be used to interpret the outcome of the SCT, rather than the visual lateralization scores [58]. There is sufficient evidence for good validity of the SCT [59-61].

Van Heugten et al. developed a diagnostic tool for apraxia in stroke, based on an existing instrument [62]. This Apraxia test, differentiating between apraxia and non-apraxia, involves demonstration of object use and imitations of gestures. It has good validity and reliability [62,63].

The SAN (Stichting Afasie Nederland = Dutch Aphasia Foundation) score is used to quantify communicative impairment in stroke patients and is part of the Aachen Aphasia Test (AAT) [64]. The SAN score is an ordinal 7-point scale with '1' indicating no communication possible and '7' indicating normal language skills [65].

#### • Behavior

The NeuroPsychiatric Inventory (NPI), originally developed for dementia patients [66], gives a global impression of behavioral problems and is applicable in other patient groups as well. The NPI comprises 12 categories of problem behaviors: delusions, hallucinations, agitation/aggression, depression, anxiety, euphoria, disinhibition, irritability/lability, apathy, aberrant motor activity, sleeping disorder and eating disorder. If the interviewed person, either a nurse in the NPI-Nursing Home (NPI-NH) version or a partner or close relative in the questionnaire version (NPI-q), positively answers the screening question, both frequency and severity (only in the NPI-NH version) are determined. The NPI closes each category with enquiring about emotional burden. The NPI is a valid and reliable instrument [66], has been translated into Dutch, and has previously been used in stroke research [67,68].

The eight item version of the Geriatric Depression Scale (GDS-8) is a shortened patient-friendly test derived from the GDS-15 version, and has been developed specifically for the nursing home population [69]. It indicates the presence of depression at a cut-off of 3 out of 8.

#### • Quality of life

The RAND- 36, developed to measure health related quality of life in chronically ill patients, comprises eight dimensions: physical functioning, role limitations due to physical health problems, bodily pain, general health, vitality, social functioning, role limitations due to emotional problems, and general mental health. It also contains an additional item about perceived health change [70]. The item scores of all dimensions need to be recoded according to the RAND health sciences program standards [71]. The RAND-36 has been translated into Dutch by van der Zee et al., and was found to be a valid, reliable, and sensitive measurement of general health [72].

#### • Caregivers

The Dartmouth COOP Functional Health Assessment Charts/WONCA (COOP/WONCA) subscales [73-75] physical fitness, daily activities, feelings and overall health are used to measure proxy's functional status. Each subscale consists of a short title and an illustrated five-point response scale: scores 16 and up are indicative of high strain [56].

The Caregiver Strain Index (CSI) is only used after discharge from the nursing home, when participation level of the patient plays a key role [76]. Optimal reintegration reduces the experienced strain of the caregivers. The CSI consists of 13 'yes' and 'no' questions, is an easy used instrument to identify strain, and shows validity [77]. A score of 7 or more positive responses indicates a high level of strain [78]. The CSI has been used in research on various diseases [79-81].

### Data analysis

All data is processed using the Statistical Package for Social Science 16.0 (SPSS 16.0). Different techniques will be used to analyze the data, depending on the research question.

• Descriptive analysis will be used for general patient characteristics, disease characteristics, treatment, successfulness of rehabilitation, and functional outcomes.

• Univariate analyses, parametric as well as non-parametric, will be performed for identifying the demographic and clinical factors that are associated with successful rehabilitation (p < 0.1).

• Associated factors will then be tested in a multivariate logistic regression analysis to determine their unique contribution and overall explained variance of successfulness of rehabilitation.

### Power

The required sample size was estimated using the rule of thumb according to Peduzzi et al. [82]: At least 10 patients per factor in the smallest group, in the case of a dichotomous outcome. Based on our experience, approximately 35% of the stroke patients, admitted to nursing homes for rehabilitation, cannot be discharged to an independent living situation. When testing a maximum of seven factors in the multivariate model, 70 patients need to be included in the smallest group (35%). Consequently, a total of 200 stroke patients will be included.

## Discussion

To our knowledge, this is the first large study that focuses on the determinants of success of geriatric stroke patients admitted to nursing homes. It will provide more detailed information about the factors that are uniquely associated to the successfulness of geriatric stroke rehabilitation and that can, thus, be used in building a clinical prediction model of discharge destination from nursing homes.

All selected outcome measures have proven to be reliable and valid, or are recommended by the Netherlands Heart Foundation.

Because legally incapable patients are excluded from this study, its external validity may be slightly affected. Therefore, general patient characteristics of the excluded patients are registered and compared to those of the included patients. Besides age, length of stay in the nursing home, and discharge destination are recorded to compare both groups. This multicenter research uses multidisciplinary teams to collect the data over a period of two-and-a-half years and, thus, may suffer from some measurement inaccuracies. To minimize such inaccuracies, over 90 people working in 15 Dutch nursing homes received the same instructions about performing the outcome measures during collective meetings before the start of the study. To ensure the quality of data collection during the study, each nursing home has 2 to 3 specially assigned professionals who maintain contact with the main researchers. In addition, a newsletter is provided every 6-8 weeks to keep everybody involved, informed, and motivated with regard to the progress of the study.

## Competing interests

The authors declare that they have no competing interests.

## Authors' contributions

MS and BB are the primary investigators of the GRAMPS study, they designed the study and wrote the manuscript. The collected data will be processed and analyzed by MS and BB. SZ will help in the analysis of the data, and he participated in writing the manuscript. FV participated in the design of the study, and he reviewed this study protocol. AG participated in designing this study, writing the manuscript, and he will help in the analysis of the data. RK participated in the design of the study, and writing the manuscript, and he will help with the analysis of the data. All authors have given final approval of the version to be published.

## Pre-publication history

The pre-publication history for this paper can be accessed here:

http://www.biomedcentral.com/1471-2318/10/15/prepub
